# Improvement of yeast tolerance to acetic acid through Haa1 transcription factor engineering: towards the underlying mechanisms

**DOI:** 10.1186/s12934-016-0621-5

**Published:** 2017-01-09

**Authors:** Steve Swinnen, Sílvia F. Henriques, Ranjan Shrestha, Ping-Wei Ho, Isabel Sá-Correia, Elke Nevoigt

**Affiliations:** 1 Department of Life Sciences and Chemistry, Jacobs University Bremen gGmbH, Campus Ring 1, 28759 Bremen, Germany; 2 Department of Bioengineering, Institute for Bioengineering and Biosciences, Instituto Superior Técnico, Universidade de Lisboa, 1049-001 Lisbon, Portugal

**Keywords:** *Saccharomyces cerevisiae*, Acetic acid tolerance, Response and adaptation to acetic acid, Haa1, Transcription factor engineering

## Abstract

**Background:**

Besides being a major regulator of the response to acetic acid in *Saccharomyces cerevisiae,* the transcription factor Haa1 is an important determinant of the tolerance to this acid. The engineering of Haa1 either by overexpression or mutagenesis has therefore been considered to be a promising avenue towards the construction of more robust strains with improved acetic acid tolerance.

**Results:**

By applying the concept of global transcription machinery engineering to the regulon-specific transcription factor Haa1, a mutant allele containing two point mutations could be selected that resulted in a significantly higher acetic acid tolerance as compared to the wild-type allele. The level of improvement obtained was comparable to the level obtained by overexpression of *HAA1*, which was achieved by introduction of a second copy of the native *HAA1* gene. Dissection of the contribution of the two point mutations to the phenotype showed that the major improvement was caused by an amino acid exchange at position 135 (serine to phenylalanine). In order to further study the mechanisms underlying the tolerance phenotype, Haa1 translocation and transcriptional activation of Haa1 target genes was compared between Haa1 mutant, overproduction and wild-type strains. While the rapid Haa1 translocation from the cytosol to the nucleus in response to acetic acid was not affected in the Haa1^S135F^ mutant strain, the levels of transcriptional activation of four selected Haa1-target genes by acetic acid were significantly higher in cells of the mutant strain as compared to cells of the wild-type strain. Interestingly, the time-course of transcriptional activation in response to acetic acid was comparable for the mutant and wild-type strain whereas the maximum mRNA levels obtained correlate with each strain’s tolerance level.

**Conclusion:**

Our data confirms that engineering of the regulon-specific transcription factor Haa1 allows the improvement of acetic acid tolerance in *S. cerevisiae*. It was also shown that the beneficial S135F mutation identified in the current work did not lead to an increase of *HAA1* transcript level, suggesting that an altered protein structure of the Haa1^S135F^ mutant protein led to an increased recruitment of the transcription machinery to Haa1 target genes.

## Background

The yeast *Saccharomyces cerevisiae* is a favorite microorganism in industrial biotechnology, mainly due to its robustness under real process conditions, its tolerance to low pH, and its impressive accessibility and versatility for metabolic engineering. Although bioethanol is still the largest-scale product in industrial biotechnology, research and development programs on engineering *S. cerevisiae* for the production of other valuable compounds are underway, and production processes for a few biofuels, bulk and fine chemicals have already been commercialized. Examples of the latter are isobutanol, farnesene, succinic acid and resveratrol [1].

**Fig. 1:**
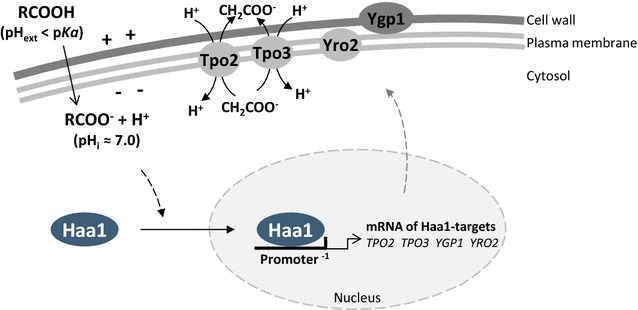
Haa1-induced activation of the four selected target genes within the Haa1-regulon that are relevant in the current study and involved in the adaptation to acetic acid stress. Upon exposure of cells to acetic acid, Haa1 binds to the promoter region of its target genes and thereby regulates their expression. We refer to the background section for more detailed information regarding the function of the depicted target genes

High tolerance to acetic acid is a favorable phenotype for microorganisms used in industrial biotechnology, in particular in the light of using renewable feedstocks such as lignocellulosic biomass. In fact, acetic acid in lignocellulosic hydrolysates originates from the acetyl groups in hemicelluloses, which are released after the essential pre-treatment step and the subsequent enzymatic breakdown of the polysaccharides into fermentable sugars [2, 3]. At the low pH values usually used in fermentation processes, acetic acid is mainly present in its protonated form, which can freely diffuse across the plasma membrane into the cell. Once inside the cytosol at near-neutral pH, acetic acid dissociates into a proton and the acetate counterion, both accumulating in the cytosol (Fig. 1). The cytosolic acidification together with the induced permeabilization of the plasma membrane is assumed to lead to the dissipation of the proton gradient across the plasma membrane required for secondary transport, thereby inhibiting or completely blocking metabolic activity depending on the level of the stress [4]. Swinnen et al. [5] and Fernandez-Nino et al. [6] have recently shown that only a fraction of cells in a population is able to recover from the stressful conditions evoked by acetic acid and resume proliferation; the size of this fraction depends both on the concentration of the acid and the genetic background of the strain. Notably, most previous studies (including those mentioned in the next paragraphs) have been conducted in comparably low acetic acid concentrations that allowed the majority of cells to resume proliferation after acetic acid exposure but still significantly influenced the duration of the latency phase.

In order to investigate the complex global response of *S. cerevisiae* to acetic acid exposure, the *S. cerevisiae* deletion strain collection has been screened to identify single-gene deletions that alter susceptibility to acetic acid [4], and a number of transcriptomic analyses have been conducted in order to study the transcriptional response of this organism to acetic acid exposure [7–13]. In this way, the transcription factor Haa1 has been found to be a major regulator of the yeast’s response to acetic acid. It is involved in the activation of approximately 80% of the acetic acid-responsive genes, which include genes encoding protein kinases, multidrug resistance transporters, proteins involved in lipid metabolism and nucleic acid processing, and proteins of unknown function [7]. Haa1 seems to be also an important determinant of the yeast’s tolerance to acetic acid stress, as the deletion of a significant number of genes of the Haa1-regulon was shown to result in increased susceptibility to acetic acid [7]. Moreover, several recent studies have shown that overexpression of *HAA1* improves yeast’s tolerance to high concentrations of acetic and lactic acid [14–17]. Given that the deletion of the *HAA1* gene was found to lead to a higher accumulation of labeled acetic acid in stressed cells [18], the role of Haa1 is, at least partially, related with its involvement in the reduction of the intracellular acetate concentration. It is thought that the latter is mediated by Haa1-induced activation of *TPO2* and *TPO3* genes encoding two drug/H^+^-antiporters that presumably mediate the active efflux of the acetic acid counterion [18] (Fig. 1).

Haa1 binds the promoter region of its target genes through the recognition of the minimal functional binding motif Haa1-responsive element (HRE) 5′-(G/C)(A/C)GG(G/C)G-3′ [19]. The Haa1/HRE recognition process was examined using a new Quartz Crystal Microbalance (QCM) analytical method and a transmission line model (TLM) algorithm to characterize the mechanical properties of the Haa1/DNA complex and the effect of single point mutations on this recognition process [20, 21]. Among all genes upregulated by acetic acid exposure that are dependent on Haa1 expression, 55% contain the HRE motif in the promoter region, and are considered to be direct targets of Haa1. Among the latter genes are the above-mentioned *TPO2* and *TPO3* genes encoding two major facilitator superfamily transporters required for multidrug resistance (MFS-MDR), previously involved in polyamine resistance and efflux [22]; the *YGP1* gene encoding a cell-wall glycoprotein, expressed under nutrient starvation conditions [23]; and the *YRO2* gene encoding a protein homolog to Hsp30 up-regulated upon potassium starvation and required for yeast tolerance to acetic acid stress [24] (Fig. 1). The remaining genes that do not contain the HRE motif in the promoter region are presumed to be indirect targets of Haa1 [7]. In this respect, Haa1 directly regulates the transcription factor encoding genes *MSN4*, *NRG1, FKH2, STP4* and *COM2* in the presence of acetic acid, while their respective gene products regulate the expression of several other genes, which are thus Haa1-indirect targets via a complex regulatory network [7].

In response to inhibitory concentrations of lactic acid, Haa1 was found to rapidly migrate from the cytoplasm to the nucleus suggesting that the biological activity of the transcription factor is dependent on its subcellular localization [14]. The Haa1 translocation is accompanied by a decrease in the phosphorylation level of the protein indicating that the transcription factor is activated upon dephosphorylation [14]. The mediated export of Haa1 by the exportin Msn5, found to directly interact with Haa1 in a large-scale yeast two-hybrid screening [25], was confirmed in *msn5*Δ cells harboring GFP-fused Haa1 [14]. The cells accumulated Haa1 in the nucleus even in the absence of lactic acid, suggesting that Haa1 constantly shuffles between the cytoplasm and the nucleus, being retained in the nucleus upon lactic acid stress [14]. No equivalent information is currently available for Haa1 translocation in response to acetic acid stress.

Inspired by the success of global transcription machinery engineering (gTME) in improving industrially-relevant multifactorial properties of microorganisms [26], we thought about a similar approach for improving acetic acid tolerance of *S. cerevisiae* but focusing on the regulon-specific transcription factor Haa1 rather than on a global transcription factor. After error-prone PCR of the *HAA1* coding sequence, a highly tolerant mutant allele carrying two point mutations was selected for further study of the mechanisms underlying the tolerance phenotype. A strain harboring a second copy of the native *HAA1* expression cassette was also constructed and included in the study. We investigated the impact of the modifications on the fraction of cells resuming proliferation and on the mRNA levels from selected Haa1-target genes. We also studied the subcellular translocation of Haa1 wild-type and mutant proteins during early response to acetic acid stress.

## Methods

### Strains and cultivation conditions

All *S. cerevisiae* strains used in this study are listed in Table 1. *S. cerevisiae* cells were routinely maintained on solid YPD medium containing 10 g L^−1^ yeast extract, 20 g L^−1^ peptone, 20 g L^−1^ glucose and 15 g L^−1^ agar, or on solid synthetic medium containing 5 g L^−1^ (NH_4_)_2_SO_4_, 3 g L^−1^ KH_2_PO_4_, 0.5 g L^−1^ MgSO_4_·7H_2_O, 20 g L^−1^ glucose, 20 g L^−1^ agar and appropriate amounts of trace elements and vitamins according to Verduyn et al. [27]. Acetic acid tolerance assays were performed in synthetic medium containing acetic acid at the indicated concentrations, and pH was adjusted to 4.5 with 4 M KOH. *S. cerevisiae* cells were routinely cultivated in a static incubator at 30 °C, or in an orbital shaker at 200 rpm and 30 °C.

**Table 1:** *S. cerevisiae* strains used in this study

Strain name	Genotype	References
BY4741 *haa1*Δ	*MAT*a *his3*Δ*1 leu2*Δ*0 met15*Δ*0 ura3*Δ*0 HAA1*::kanMX	[43]
CEN.PK113-13D	*MAT*α *MAL2*-*8* ^*c*^ *SUC2 ura3*-*52*	[44]
CEN.PK113-13D *haa1*Δ	*MAT*α *MAL2*-*8* ^*c*^ *SUC2 ura3*-*52 HAA1*::kanMX	This study
CEN.PK113-7D	*MAT*a *MAL2*-*8* ^*c*^ *SUC2*	[44]
CEN.PK113-7D Haa1^S135F^	*MAT*a *MAL2*-*8* ^*c*^ *SUC2 HAA1*::*HAA1* ^*C404T*^	This study
CEN.PK113-7D Haa1^Truncated^	*MAT*a *MAL2*-*8* ^*c*^ *SUC2 HAA1*::*HAA1* ^*G1447T*^	This study
CEN.PK113-7D Haa1^S135F_Truncated^	*MAT*a *MAL2*-*8* ^*c*^ *SUC2 HAA1*::*HAA1* ^*C404T_G1447T*^	This study
CEN.PK113-7D Haa1^OE^	*MAT*a *MAL2*-*8* ^*c*^ *SUC2 YGLCτ3*::*HAA1*	This study

*Escherichia coli* cells (DH5α) were cultivated in lysogeny broth (LB) medium containing 10 g L^−1^ yeast extract, 20 g L^−1^ peptone and 10 g L^−1^ NaCl. For solid medium, 15 g L^−1^ agar was added. *E. coli* cells were cultivated in a static incubator at 37 °C, or in an orbital shaker at 250 rpm and 37 °C.

### General molecular biology methods

*Saccharomyces cerevisiae* transformations were performed using the lithium acetate method described by Gietz et al. [28], and *E. coli* transformations using the CaCl_2_ and heat shock method described by Sambrook and Russell [29]. Genomic DNA was extracted from *S. cerevisiae* cells using a mixture of phenol, chloroform and isoamyl-alcohol according to Hoffman and Winston [30]. Polymerase chain reaction (PCR) was performed with TaKaRa Ex Taq Polymerase (Merck KGaA, Darmstadt, Germany) for diagnostic purposes, and Phusion High-Fidelity DNA Polymerase (Thermo Fisher Scientific, MA, USA) for genetic manipulation and sequencing purposes. Sequencing was carried out using the dideoxy chain-termination method [31] at GATC Biotech AG (Konstanz, Germany). Sequences were analyzed with Sequencher® version 5.3 sequence analysis software (Gene Codes Corporation, Ann Arbor, USA; available at http://genecodes.com) and SnapGene® software (GSL Biotech; available at http://www.snapgene.com/).

### Deletion of *HAA1* in strain CEN.PK113-13D

The *HAA1* gene was deleted in strain CEN.PK113-13D by using a cassette conferring resistance to G418 as a selectable trait. The deletion cassette was PCR-amplified from genomic DNA of strain BY4741 *haa1*Δ with primers HAA1_del_fw and HAA1_del_rv (Table 2). The deletion cassette was purified from the reaction mixture by using a PCR purification kit (Qiagen, Hilden, Germany), and subsequently used for transformation of the strain CEN.PK113-13D. Transformants were selected on YD containing 100 mg L^−1^ G418. The correct integration of the deletion cassette was checked by PCR with primers HAA1_del_control_fw and HAA1_del_control_rv (Table 2).

**Table 2:** Primers used in this study

Primer name	Sequence (5′–3′)
HAA1_del_fw	ACAGCACCAGCACTTGATTG
HAA1_del_rv	ATGGTCTTACTCTCTGATACC
HAA1_del_control_fw	ACAGAGTCGTGCATTTCCAC
HAA1_del_control_rv	CCATGAGTGACGACTGAATC
HAA1_BamHI_fw	CAGTGGATCCCTCTATGAGAAGAACCCACG
HAA1_BamHI_rv	CAGTGGATCCTACACAACAAACTACGCAAGG
HAA1_epPCR_fw	AAAAAGGAAACAAAAGTATAGAAAAAAAAAACCTAAAAAATAATG
HAA1_epPCR_rv	AAACTACAGTTACAGAGAAGCAAGAGACGAAAAGCAAATTTATCA
HAA1_C404T_GIN11_fw	CTGGCACAGAAAGCCAAAGAAGAAGCAAGAGCTAAAGCCAATCGGAACCCTAAAGGGAGC
HAA1_G1447T_GIN11_fw	TTTACAGATTCATCGTCGATTTCAACGCTTTCCCGTGCAAATCGGAACCCTAAAGGGAGC
HAA1_GIN11_rv	GGTACCAGGAAATGAAAGCG
HAA1_kanMX_fw	GGGCTGCAGGAATTCGATATCAAGCTTATCGATACCGTCGCAGCTGAAGCTTCGTACGC
HAA1_C404T_kanMX_rv	TCAGCTTTCGCTGGCAAGCTTACCGAACTATCTTGCCAGTGCATAGGCCACTAGTGGATCTG
HAA1_G1447T_kanMX_rv	CAGCTAGGTTTGAAGGGTCCATCATCATATTTGCTATCGAGCATAGGCCACTAGTGGATCTG
HAA1_control_fw	ACAGCACCAGCACTTGATTG
GIN11_rv	CTGAAACGCAGATGTGCCTC
HAA1_C404T_fw	TGTGAGAGGTGCATAAGAGG
HAA1_C404T_rv	CCAATCGTAGACCAAAGAGC
HAA1_G1447T_fw	GCTCTTTGGTCTACGATTGG
HAA1_G1447T_rv	TGGTTCATCTCGTCTGGTAC
HAA1_OE_GIN11_fw	AGCTTCTTTCCACGAAAGAAATAGTGTAAGTTAGAGGTACATCGGAACCCTAAAGGGAGC
HAA1_OE_kanMX_rv	ATTAAGAAGCAAATACCTTCCTGTTGCTTGATTTGCCCTGGCATAGGCCACTAGTGGATCTG
HAA1_OE_fw	ATGTAGTTCAACTTCTATGAATGCTCGGCGATACGATATGCTCTATGAGAAGAACCCACG
HAA1_OE_rv	AAGGCTCATTTCCATGATGGGGTCACAATTATTATCGCACTACACAACAAACTACGCAAGG
HAA1_OE_control_fw	GCAATCTCGACGATCAACTG
HAA1_OE_control_rv	AAGAACCAGAATGGCAGGAC
HAA1_GFP-NAT_fw	CGATCAAGGATTTGCGGATTTGGATAATTTCATGTCTTCGTTA CGG ATC CCC GGG TTA ATT AA
HAA1_GFP-NAT_rv	CTACAGTTACAGAGAAGCAAGAGACGAAAAGCAAATTTA TCAGAATTCGAGCTCGTTTAAAC
HAA1_STOP483_GFP-NAT_fw	CTTTGACACCGAGTTTTATGGATATTCCCGAAAAAGAAAGA CGG ATC CCC GGG TTA ATT AA
HAA1_STOP483_GFP-NAT_rv	CTGTCAGTAATGTAATTGGATGATGGCGATCTTTCCGTTTA TCA GAATTCGAGCTCGTTTAAAC
ACT1_RT_fw	CTCCACCACTGCTGAAAGAGAA
ACT1_RT_rv	CCAAGGCGACGTAACATAGTTTT
HAA1_RT_fw	TCGTGTGGGCGAAGTTAGC
HAA1_RT_rv	CCAACCCCATCAATGTCAGAA
TPO2_RT_fw	TGAGTGATCAAGAATCTGTTG
TPO2_RT_rv	CGGTACGGTTCAATTGCTTT
TPO3_RT_fw	TTGTGACTGGCGATCCAGAA
TPO3_RT_rv	ACTCCAACGGATCCATGCA
YGP1_RT_fw	TGTACAATGTTGCCCGTGTTG
YGP1_RT_rv	GGCACCGGCGGATGA
YRO2_RT_fw	TGGATCCCAGTCAGAGCAAAGT
YRO2_RT_rv	ACCTGGGTGCTCCTTTTGG
FPS1_RT_fw	ATCTGGGCCCACGTCTTG
FPS1_RT_rv	ATGCACCCAAAGCATTTTATGA

### Construction of plasmid pRS416-*HAA1*

The *HAA1* gene (containing the *HAA1* coding sequence together with 1086 bp upstream and 412 bp downstream of the start and stop codon, respectively) was PCR-amplified from genomic DNA of strain CEN.PK113-13D with primers HAA1_BamHI_fw and HAA1_BamHI_rv (Table 2). Each of the two primers contained at its 3′ terminal end a sequence that is complementary to a region flanking the *HAA1* gene, and at its 5′ terminal end the recognition site for the restriction enzyme *Bam*HI. The amplified fragment was purified from the reaction mixture by using a PCR purification kit, and subsequently digested with *Bam*HI (Thermo Fisher Scientific, MA, USA). The *HAA1* gene was then ligated into the *Bam*HI site of the dephosphorylated low copy (CEN/ARS) plasmid pRS416 [32] using T4 DNA ligase (Thermo Fisher Scientific, MA, USA). The ligation mixture was used to transform chemically competent *E. coli* cells, after which transformants were selected on LB medium containing 100 mg L^−1^ ampicilin. Plasmids were subsequently extracted from *E. coli* cells by using a commercial miniprep kit (Qiagen, Hilden, Germany). The correct integration of the *HAA1* gene into the plasmid pRS416 was checked by restriction analysis, and the *HAA1* gene sequence was verified by sequencing.

### Construction of the *HAA1* mutant library

A library of mutant versions of the *HAA1* coding sequence was constructed using the GeneMorph II Random Mutagenesis kit from Stratagene (California, US). At first, mutant alleles of the *HAA1* coding sequence were created via error-prone PCR using plasmid pRS416-*HAA1* as a template. The forward and reverse primers (HAA1_epPCR_fw and HAA1_epPCR_rv; Table 2) contained at their very 3′ terminal end the start or stop codon of the *HAA1* gene, respectively, which implies that mutations were only introduced in the sequences between start and stop codon. The 50-µL PCR mixtures contained 5 µL of Mutazyme II reaction buffer (10×), 1 µL of Mutazyme II DNA polymerase (2.5 U µL^−1^), 1 µL of both forward and reverse primer (10 pmol µL^−1^ each primer), 1 µL of dNTP mix (10 mM each dNTP) and 1 µL of template DNA. In total nine PCR mixtures were prepared containing three different amounts of the *HAA1* coding sequence (50, 250, and 750 ng) according to the kit’s guidelines. The following cycling parameters were used: 2 min of initial denaturation at 95 °C, and 30 cycles comprising a 30-s denaturation step at 95 °C, a 30-s annealing step at 66 °C, and a 3-min elongation step at 72 °C. The final elongation step was performed at 72 °C for 10 min. After PCR, the amplified fragments were isolated from each of the nine PCR mixtures by extraction from an agarose gel using a gel extraction kit (Qiagen, Hilden, Germany). DNA concentrations were determined using the NanoDrop 1000 spectrophotometer (Thermo Fisher Scientific, Delaware, USA), after which equal amounts of the individually obtained PCR products were combined.

In order to replace the wild-type *HAA1* coding sequence in the plasmid pRS416-*HAA1* by the mutant *HAA1* alleles, the plasmid was first digested with restriction enzymes *Eco*NI and *Bsp*EI (Thermo Fisher Scientific, MA, USA), which removed most part of the *HAA1* coding sequence (*Eco*NI cuts at 33 bp downstream of the start codon, and *Bsp*EI at 122 bp upstream of the stop codon), and subsequently recovered from the restriction mixture by extraction from an agarose gel. Appropriate amounts of the digested pRS416-*HAA1* plasmid and PCR products of the mutant *HAA1* alleles in a molar ratio of 1–5 (100 and 147 ng of DNA, respectively) were then used to transform strain CEN.PK113-13D *haa1*Δ. The flanking sequences of the PCR products homologous to the *HAA1* promoter and terminator allowed in vivo recombinatorial cloning. The transformation mixtures were afterwards spread on 23 square Petri dishes (120 × 120 mm) with solid synthetic medium. After 4 days of incubation at 30 °C, 2000–5000 single cell colonies were obtained on each plate. A parallel transformation only using the digested plasmid resulted in about 200–300 colonies indicating that less than 1 in 10 colonies reflecting the *HAA1* mutant library might contain an insert-less plasmid. To obtain the total *HAA1* mutant library the cells from each of the 23 plates were washed off with 1 mL of sterile water, and subsequently mixed together in one tube. Aliquots of 1 mL were mixed with 200 µL of glycerol and stored at −80 °C.

### Enrichment of the *HAA1* mutant library for alleles that confer improved acetic acid tolerance

For pre-culture, 5 mL of synthetic medium in a glass tube were inoculated with 50 µL of the *HAA1* mutant library glycerol stock. The cells were cultivated overnight in an orbital shaker at 200 rpm and 30 °C. The pre-culture was used to inoculate 50 mL of fresh synthetic medium in a shake flask to an optical density (OD_600_) of 0.2. This culture was cultivated under the same conditions as the pre-culture for 6–8 h until mid-exponential phase was reached (i.e. OD_600_ between 1.0 and 1.5). An appropriate amount of cells to obtain an OD_600_ of 0.2 in 50 mL was pelleted by centrifugation (800*g* for 5 min), and resuspended in 50 mL of synthetic medium containing 200 mM acetic acid at pH 4.5. This culture was cultivated until early stationary phase was reached. At this time point, a glycerol stock of the culture, which is supposed to be enriched in acetic acid tolerant mutants, was prepared and stored at −80 °C. The glycerol stock was used to inoculate a new pre-culture, and the above-described enrichment procedure was repeated for additional three rounds.

### Re-transformation of strain CEN.PK113-13D *haa1*Δ with plasmids isolated from single cell colonies obtained after the enrichment procedure

After the fourth round of enrichment, an aliquot of the cell culture was streaked on solid synthetic medium to obtain single cell colonies. Plasmid DNA was extracted from several of these colonies according to the method described by Singh and Weil [33] with some modifications. In particular, cells originating from a single cell colony were used to inoculate 50 mL of synthetic medium, and subsequently cultivated overnight in an orbital shaker. The cells were then pelleted by centrifugation at 1811*g* for 5 min, and resuspended in 1 mL of P1 buffer from the miniprep kit. The cell suspension was transferred to a microcentrifuge tube, and approximately 1 g of acid-washed glass beads (diameter of 425–600 µm) was added. The cells were subsequently lysed by vortexing the tube vigorously for 5 min. After the glass beads had settled, 250 µL of the cell lysate were transferred to a new microcentrifuge tube, and further treated according to the guidelines given in the kit’s manual. The plasmid DNA was afterwards eluted from the miniprep column with 100 µL of water, and used to transform strain CEN.PK113-13D *haa1*Δ.

### Targeted introduction of single nucleotide mutations into the genome

Introduction of the single nucleotide mutations C404T and G1447T in the native *HAA1* gene of strain CEN.PK113-7D was achieved in two steps using markers for selection and subsequent counterselection. In the first step, a part of the *HAA1* gene (containing the locus with the mutation to be introduced) was replaced by a cassette containing the kanMX gene and a galactose-inducible growth inhibitory sequence (referred to as *GALp*-*GIN11M86*). The *GALp*-*GIN11M86* sequence was amplified from plasmid pGG119 [34] by PCR using primers HAA1_(mutation)_GIN11_fw and HAA1_GIN11_rv (Table 2), while the kanMX gene was amplified from plasmid pUG6 using primers HAA1_kanMX_fw and HAA1_(mutation)_kanMX_rv (Table 2). Primers HAA1_GIN11_rv and HAA1_kanMX_fw were designed to result in a complementary sequence between the two generated PCR products downstream of the *GALp*-*GIN11M86* sequence and upstream of the kanMX gene, so that both cassettes could be assembled by homologous recombination upon co-transformation. Primers HAA1_(mutation)_GIN11_fw and HAA1_(mutation)_kanMX_rv contained at their 5′ terminal ends 40-bp sequences complementary to regions upstream and downstream of the *HAA1* sequence to be exchanged in the genome. Both PCRs were performed using Phusion High-Fidelity DNA Polymerase according to the manufacturer’s guidelines. After purification of the PCR products, strain CEN.PK113-7D was co-transformed with equimolar amounts of each product, and transformants were selected on YD medium containing 100 mg L^−1^ G418. The correct integration of the cassette was verified by PCR using primers HAA1_control_fw and GIN11_rv (Table 2).

In the second step, the kanMX/*GALp*-*GIN11M86* cassette in strain CEN.PK113-7D was removed by transformation of the strain with a PCR product containing the *HAA1* allele with the respective mutation, flanked by sequences homologous to regions upstream and downstream of the chromosomal position of the kanMX/*GALp*-*GIN11M86* cassette. The PCR was performed with Phusion High-Fidelity DNA Polymerase using primers HAA1_(mutation)_fw and HAA1_(mutation)_rv (Table 2) and plasmid pRS416-*HAA1* ^*C404T_G1447T*^ as a template. After transformation, cells were plated on solid synthetic medium containing 2% galactose as the sole carbon source for induction of the growth inhibitory sequence *GIN11M86*. Correct integration of the single nucleotide mutations into the genome was verified by PCR and sequencing.

### Overexpression of *HAA1* in strain CEN.PK113-7D

Overexpression of the *HAA1* gene in strain CEN.PK113-7D was achieved by integration of an additional CEN.PK113-7D wild-type *HAA1* allele (including native promoter and terminator) at the YGLCτ3 site on chromosome VII (integration site 8 according to Flagfeldt et al. [35]). The method used was similar to the one described for the introduction of single nucleotide mutations into the genome; only the primers used were different. For the first step, the *GALp*-*GIN11M86* sequence was amplified using primers HAA1_OE_GIN11_fw and HAA1_GIN11_rv (Table 2), while the kanMX gene was amplified using primers HAA1_kanMX_fw and HAA1_OE_kanMX_rv (Table 2). For the second step, the *HAA1* allele was amplified from genomic DNA of strain CEN.PK113-7D using primers HAA1_OE_fw and HAA1_OE_rv (Table 2). Correct integration of the gene was verified by PCR using primers HAA1_OE_control_fw and HAA1_OE_control_rv (Table 2).

### Quantitative analysis of acetic acid tolerance in liquid medium using the Growth Profiler 1152, and on solid medium

For pre-culture, 3 mL of synthetic medium were inoculated with cells originating from a single cell colony on plate. The cells were cultivated overnight in an orbital shaker at 200 rpm and 30 °C. The pre-culture was used to inoculate 3 mL of fresh synthetic medium to an OD_600_ of 0.2. This culture (referred to as the intermediate culture) was cultivated under the same conditions as the pre-culture until mid-exponential phase was reached (i.e. OD_600_ between 1.0 and 1.5). For acetic acid tolerance assays in liquid medium, an appropriate amount of cells from the intermediate culture to obtain an OD_600_ of 0.2 in 5 mL was collected by centrifugation (800*g* for 5 min), and subsequently resuspended in 5 mL of synthetic medium either with or without acetic acid (pH 4.5). An aliquot of 750 µL from the latter culture was transferred immediately into a well of a white Krystal 24-well clear bottom microplate (Porvair Sciences, Leatherhead, UK). Growth was recorded using the Growth Profiler 1152 (Enzyscreen, Haarlem, The Netherlands) as previously described [5].

For acetic acid tolerance assays on solid medium, an appropriate amount of cells from the intermediate culture to obtain an OD_600_ of 0.2 in 1 mL was collected by centrifugation (800*g* for 5 min), and subsequently resuspended in 1 mL of synthetic medium without acetic acid and without glucose. This cell suspension was then serially diluted in the same medium to obtain dilutions of 10^−1^ to 10^−4^. An aliquot of 250 µL of each dilution was spread on solid synthetic medium either with or without acetic acid (pH 4.5). Plates were then incubated in a static incubator at 30 °C. The incubation time was 2 days for the medium without acetic acid, and 4 days for the medium with acetic acid. Dilutions resulting in colony forming units (CFU) in the range of 50–150 per plate were included for counting.

### Determination of the transcription profiles of *HAA1* and Haa1-regulated genes in cells exposed to acetic acid

Real-time RT-PCR was performed to determine the mRNA levels from *HAA1* and Haa1-target genes *TPO2*, *TPO3*, *YGP1* and *YRO2* in cells of strain CEN.PK113-7D expressing either wild-type or mutant Haa1 proteins immediately before and at different time points after exposure to acetic acid. The *FPS1* gene was included as a negative control since its regulation is Haa1-independent, and the *ACT1* gene was included as an internal control. Cells of each strain were used to inoculate 30 mL of synthetic medium, and subsequently cultivated overnight in an orbital shaker at 200 rpm and 30 °C. This pre-culture was used to inoculate 110 mL of fresh synthetic medium, after which the culture was cultivated until an OD_600_ of 1.0–1.5 was reached. Cells from an adequate volume of culture were then collected by filtration and resuspended in 200 mL of fresh synthetic medium in order to obtain an OD_600_ of 0.5. After 20 min of incubation in an orbital shaker at 200 rpm and 30 °C, cells of 20 mL of the culture were collected by centrifugation (5000*g* for 3 min) at 4 °C, after which the cell pellet was frozen immediately in liquid nitrogen and stored at −80 °C. In parallel, acetic acid was added to the remaining culture to a final concentration of 50 mM, and cells of 20 mL of the culture were collected after 30, 60 and 120 min using the same protocol as described for the non-stressed cells. Total RNA was extracted from the frozen cell pellets by the hot phenol method [36], and treated with DNAseI (Invitrogen, Carlsbad, USA) according to the manufacture’s guidelines. In total 1 µg of the treated RNA was used in the reverse transcription step using Taqman® reverse transcription reagents (Applied Biosystems, Branchburg, USA), and 62.5 ng of the synthesized cDNA were used in the PCR amplification step using Power SYBR® Master Mix reagents (Applied Biosystems, Warrington, UK). Primers used for the amplification of the selected cDNAs were designed using the Primer Express Software (Applied Biosystems, Foster City, USA) and are listed in Table 2. The mRNA level of *ACT1* was used for normalization of the mRNA levels of all other genes. For each of the latter genes, the mRNA levels shown are relative to the level registered in Haa1 wild-type cells cultivated in the absence of acetic acid, which was set as 1.

### Subcellular localization of Haa1 in non-stressed and acetic acid stressed cells

Fusion of the green fluorescent protein (GFP) to the C-terminus of Haa1 was achieved by insertion of the GFP(S65T)-ADH1_terminator_-natMX6 cassette directly upstream of the stop codon of the *HAA1* sequence. The cassette was PCR-amplified from plasmid pFA6a-GFP(S65T)-natMX6 [37] using primers HAA1_GFP-NAT_fw and HAA1_GFP-NAT_rv (Table 2) for insertion of the cassette downstream of the wild-type *HAA1* or mutant *HAA1* ^*C404T*^ allele, or primers HAA1_STOP483_GFP-NAT_fw and HAA1_STOP483_GFP-NAT_rv (Table 2) for insertion of the cassette downstream of the mutant *HAA1* ^*G1447T*^ allele, which contains a premature stop codon. The cassettes were purified from the reaction mixtures, and subsequently used for transformation of the respective wild-type and mutant CEN.PK113-7D strains. Transformants were selected on solid YPD medium containing 100 µg mL^−1^ nourseothricin, after which the correct integration of the GFP(S65T)-ADH1_terminator_-natMX6 cassette was verified by sequencing. Notably, in the CEN.PK113-7D strain overexpressing *HAA1*, only one if the two alleles were fused with the GFP(S65T)-ADH1_terminator_-natMX6 cassette.

Cells expressing Haa1-GFP(S65T) fusions were cultivated in the same way as cells used for the determination of the transcription profiles of *HAA1* and Haa1-regulated genes. For nuclear staining of the cells, 5 µL of 4′,6-diamidino-2-phenylindole (DAPI, 1 mg mL^−1^) was added to 1 mL of cell suspension. The cells were subsequently incubated for 2 min at room temperature, collected by centrifugation (5000*g* for 30 s), resuspended in about 50 µL of supernatant, and visualized using fluorescence microscopy. Fluorescence images were captured with a cooled CCD camera (Cool SNAPFX, Roper Scientific Photometrics, Tucson, USA) coupled to a Zeiss Axioplan microscope (Carl Zeiss MicroImaging GmbH, Oberkochen, Germany) using excitation and emission filters of 365/12 and 397 nm to detect DAPI fluorescence signal, and 450–490 and 515 nm to detect GFP signal. ImageJ software was used to overlay the images.

## Results

### Identification of mutant *HAA1* alleles that improve acetic acid tolerance

**Fig. 2:**
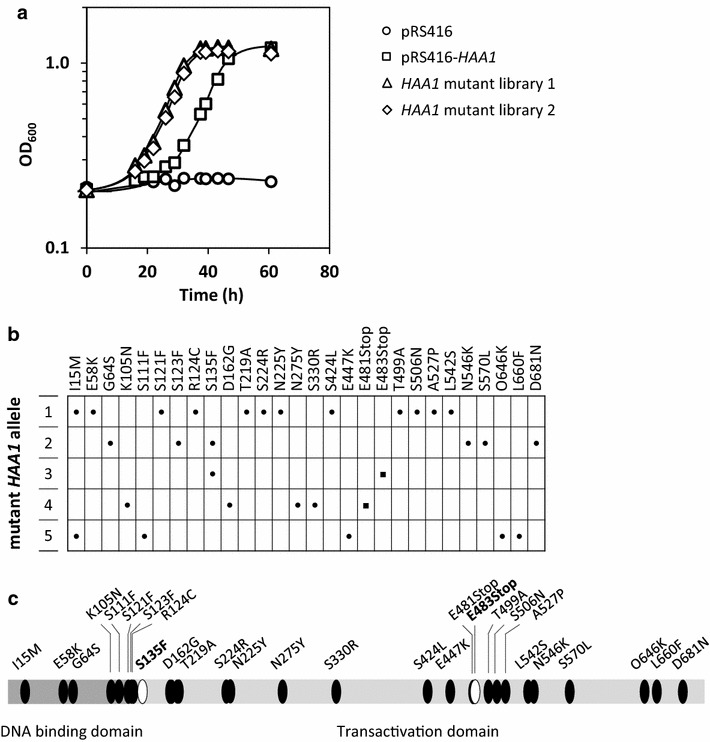
Selection of mutant *HAA1* alleles with improved tolerance to acetic acid. **a** Growth performance of two parallel cultures of an *HAA1* mutant library during the fourth round of enrichment in synthetic medium containing 200 mM acetic acid (pH 4.5) is shown. First, a CEN/ARS plasmid-based *HAA1* mutant library was generated by means of error-prone PCR and recombinatorial cloning in the *S. cerevisiae* strain CEN.PK113-13D *haa1*Δ. Two aliquots of the library were then cultivated under non-stress conditions until exponential growth phase, after which the cells were transferred to synthetic medium containing 200 mM acetic acid (pH 4.5). This procedure of alternating non-stress and acetic acid stress conditions was repeated four times. For comparison, cells of an isogenic strain containing the wild-type *HAA1* allele cloned in the same plasmid backbone (pRS416-*HAA1*) were subjected to the same enrichment procedure, and the growth performance in acetic acid containing medium during the fourth round of enrichment is shown as well. The strain CEN.PK113-13D *haa1*Δ containing the empty plasmid (pRS416) was always included as a control. **b** Modifications in protein sequence deduced from five non-redundant mutant *HAA1* alleles isolated from the enriched library cultures that significantly improved acetic acid tolerance. **c** Relative position of the identified mutations in the Haa1 DNA binding and transactivation domains

The first part of this study aimed at generating a plasmid-based library of mutant *HAA1* alleles, and isolating alleles able to improve the acetic acid tolerance of *S. cerevisiae* strain CEN.PK113-13D *haa1*Δ in comparison to the wild-type *HAA1* allele (*HAA1* ^WT^). Briefly, mutant alleles of the *HAA1* coding sequence were created by error-prone PCR, after which the PCR products were placed between the native *HAA1* promoter and terminator in the backbone of the low-copy (CEN/ARS) pRS416 plasmid by recombination-based cloning in the strain CEN.PK113-13D *haa1*Δ. The transformation resulted in more than 80,000 clones. Sequencing of plasmids isolated from 15 randomly selected clones revealed a mutation frequency between 2 and 10 non-synonymous mutations within the *HAA1* coding sequence. In order to select clones with improved acetic acid tolerance, two parallel enrichments of the library were conducted by serial batch cultivations of the cells in the presence of a relatively high concentration of acetic acid (200 mM at pH 4.5). In between each round of enrichment, the cells were brought back to non-stress conditions as described in the “Methods” section. As a control, the strain CEN.PK113-13D *haa1*Δ expressing *HAA1* ^WT^ from the same plasmid backbone was included in the enrichment experiment. After four rounds of enrichment, all cultures showed a clear improvement in acetic acid tolerance, however, the improvement was more pronounced for the library cultures than for the control culture (Fig. 2a). Aliquots from the two enriched library cultures were then streaked for single cell colonies, after which plasmid DNA was isolated from 9 clones from each library culture and introduced into a fresh CEN.PK113-13D *haa1*Δ background. Out of the 18 resulting strains, 11 showed a growth performance comparable to the enriched library cultures (data not shown), implying that the improved acetic acid tolerance of strain CEN.PK113-13D *haa1*Δ was truly caused by the expression of the mutant *HAA1* alleles. In fact, the originally isolated clones could have obtained mutations that have naturally arisen in the genomic DNA during the enrichment procedure. Sequencing of the 11 plasmids that improved acetic acid tolerance revealed five different *HAA1* alleles (Fig. 2b, c).

### Detailed characterization of the strain CEN.PK113-7D Haa1^S135F_Truncated^ and dissection of each mutation’s contribution to the phenotype

Out of the five mutant *HAA1* alleles shown in Fig. 2b, the allele with the lowest number of mutations was selected for further study. This allele contained a cytosine to thymine mutation at position 404 (resulting in a serine to phenylalanine exchange at position 135 in the protein), and a guanine to thymine mutation at position 1447 (resulting in a truncation of the protein from position 483 onwards).

**Fig. 3:**
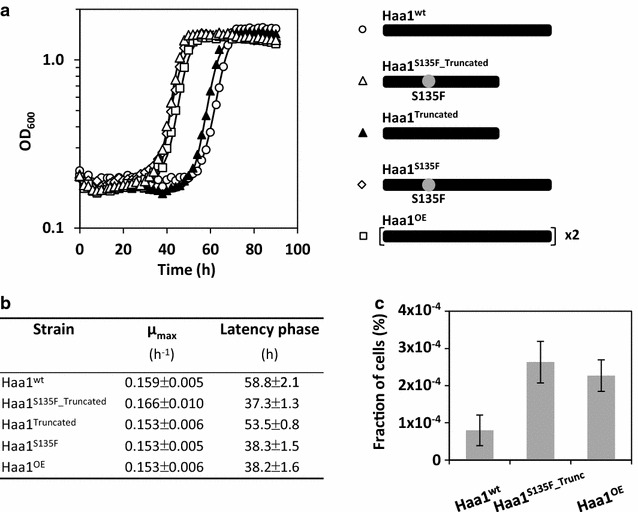
Dissection of the phenotypic contributions of each individual mutation in the identified *HAA1* mutant allele *HAA1* ^*C404T_G1447T*^ (protein Haa1^S135F_Truncated^). The effects of the modified proteins were characterized after allele swapping in strain CEN.PK113-7D, respectively. **a** Growth curves, **b** maximum specific growth rates and duration of latency phases, and **c** fraction of cells that resume proliferation are shown after shifting exponentially growing cells from medium without acetic acid to medium with 160 mM acetic acid at pH 4.5 are shown. The isogenic wild type CEN.PK113-7D (Haa1^WT^) and the strain containing a second copy of the endogenous *HAA1* expression cassette (Haa1^OE^) were also included in these experiments. Mean values and standard deviations of at least three biological replicates are shown

In order to rule out any potential side effects of the episomal expression of *HAA1* as well as of the auxotrophy of the used strain, the *HAA1* ^*C404T_G1447T*^ allele was used to replace the native *HAA1* coding sequence in the genome of the prototrophic strain CEN.PK113-7D (hereafter referred to as strain CEN.PK113-7D Haa1^S135F_Truncated^). The allele swapping was conducted in a seamless way, meaning that no markers or foreign sequences were left behind. For comparative analyses, a derivative of strain CEN.PK113-7D with a second copy of the native *HAA1* gene was also constructed (hereafter referred to as strain CEN.PK.113-7D Haa1^OE^). The acetic acid tolerance of strains CEN.PK113-7D Haa1^S135F_Truncated^ and Haa1^OE^ as compared to strain CEN.PK113-7D Haa1^WT^ was first evaluated by quantifying the effect of acetic acid on the maximum specific growth rate and duration of the latency phase. As shown in Fig. 3a, b, strains CEN.PK113-7D Haa1^S135F_Truncated^ and Haa1^OE^ did not show a significantly improved growth rate but do show a shorter latency phase in the presence of 160 mM acetic acid (pH 4.5) as compared to strain CEN.PK113-7D Haa1^WT^. Our data also shows that the expression of *HAA1* ^S135F_Truncated^ led to a slightly (reproducible but not significantly) higher acetic acid tolerance than the overexpression of *HAA1*. In addition, the fraction of cells that resume proliferation in the presence of acetic acid was quantified for each strain by counting colony forming units on solid acetic acid containing medium. This method has been previously demonstrated to properly represent the fraction of cells able to resume proliferation in liquid acetic acid containing medium [5]. Consistent with the theory and in congruence with the duration of the latency phases, both strains CEN.PK113-7D Haa1^S135F_Truncated^ and CEN.PK113-7D Haa1^OE^ showed a significantly higher fraction of proliferating cells in the presence of 160 mM acetic acid as compared to strain CEN.PK113-7D Haa1^WT^ (Fig. 3c).

After showing the effect of the expression of the *HAA1* ^C404T_G1447T^ allele on the acetic acid tolerance of strain CEN.PK113-7D, the effect of the two individual point mutations was studied in more detail. For this purpose, the two mutations were separately introduced into the genome of strain CEN.PK113-7D, generating strains CEN.PK113-7D Haa1^S135F^ and CEN.PK113-7D Haa1^Truncated^. Our data shows that both mutations have an additive effect on the acetic acid tolerance of strain CEN.PK113-7D (Fig. 3a, b); even though the amino acid exchange at position 135 exerts a by far more severe impact than the Haa1 protein truncation. In fact, the latency phase of strain CEN.PK113-7D Haa1^S135F^ was only slightly reduced in the presence of 160 mM acetic acid as compared to strain CEN.PK113-7D Haa1^S135F_Truncated^ (Fig. 3a, b).

We next sought to understand the mechanisms underlying the differences in acetic acid tolerance between the Haa1 wild-type, mutant and overproduction strains. At high acetic acid concentrations such as those used in the first part of the current work, molecular studies at the population level are hampered since only a few cells in the population resume proliferation while the remaining cells arrest growth [5, 6]. In order to rule out the effect of cell-to-cell heterogeneity, we significantly reduced the stress level to 50 mM acetic acid at pH 4.5. At this condition, all cells of strain CEN.PK113-7D have been demonstrated to resume proliferation [6], whereas differences between the strains with regard to the duration of the latency phase are still apparent (although to a lesser extent) (Fig. 5).

### Both Haa1 wild-type and mutant proteins translocate from the cytosol to the nucleus in response to acetic acid-induced stress

**Fig. 4:**
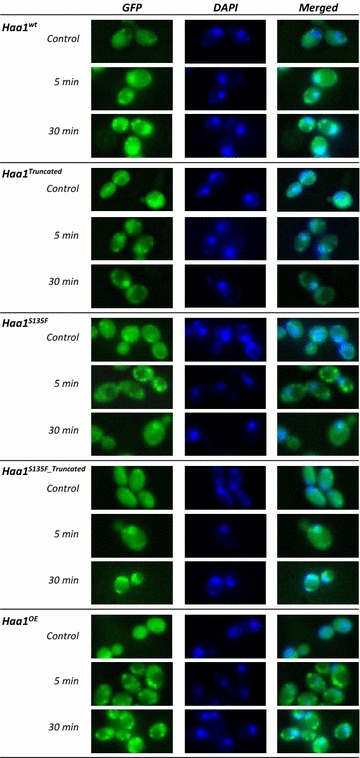
Subcellular localization of Haa1 before and after exposure of the cells to acetic acid. Results are shown for the wild-type CEN.PK113-7D strain (Haa1^WT^) and the Haa1^S135F_Truncated^, Haa1^Truncated^, Haa1^S135F^ and Haa1^OE^ CEN.PK113-7D strains. Haa1 localization was determined in cells immediately before and 5 and 30 min after the transfer to medium containing 50 mM acetic acid (pH 4.5). Cells were stained with 4′,6-diamidino-2-phenylindole (DAPI) to label nuclear DNA. Visualization was performed using fluorescence microscopy to detect DAPI and GFP fluorescence signals. ImageJ software was used to overlay the images

In a previous study it was shown that Haa1 translocates from the cytosol to the nucleus upon exposure to lactic acid [14]. In the current study it was examined whether similar Haa1 translocation also occurs upon exposure to acetic acid, and whether this translocation is affected by the different mutations. A GFP-tag was therefore inserted downstream of the wild-type and mutant *HAA1* coding sequences within the genome of the Haa1 wild-type, mutant and overproduction strains, respectively. Afterwards, the localization of Haa1 was studied in cells of all strains cultivated in the absence and presence of 50 mM acetic acid at pH 4.5. In all tested strains, Haa1 was translocated from the cytosol to the nucleus upon acetic acid exposure, and this translocation was already apparent after 5 min (Fig. 4).

### Expression of the mutant *HAA1* alleles increases transcript levels from selected Haa1-target genes

**Fig. 5:**
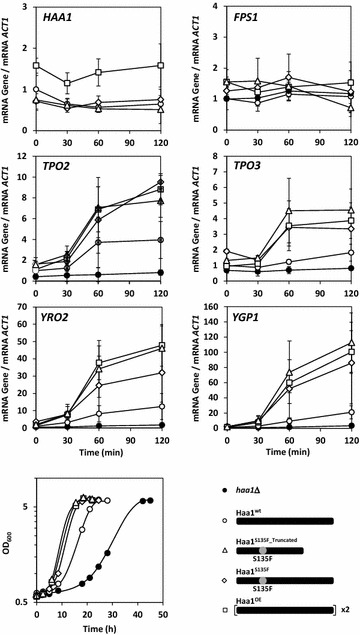
Effect of the different Haa1 modifications on the time courses of transcription levels of the *HAA1* gene, four genes belonging to the Haa1-regulon (*TPO2, TPO3, YRO2* and *YGP1*), and a reference gene (*FPS1*) whose expression has been shown to be not affected by acetic acid. Cells were cultivated in synthetic medium without acetic acid until exponential phase (time point 0) and then transferred to synthetic medium containing 50 mM acetic acid (pH 4.5). Immediately before and 30, 60 and 120 min after exposure to acetic acid, aliquots of the cell suspension were taken to determine the respective mRNA levels by real-time RT-PCR. Values represent the average of at least three independent experiments, and *ACT1* mRNA level was used as an endogenous control. The mRNA level measured in cells of the wild-type CEN.PK113-7D strain (Haa1^WT^) cultivated in the absence of acetic acid was set as 1 and used to normalize the mRNA levels determined in the other strains and conditions. The corresponding growth curves recorded by OD_600_ measurement are also shown

While overexpression of *HAA1* was expected to result in an increase of *HAA1* transcript level, the question arose as to whether expression of the mutant *HAA1* alleles would also modify *HAA1* mRNA abundance, for example, by affecting the stability of the corresponding mRNAs. For this purpose, *HAA1* transcript levels were determined in Haa1 wild-type, mutant and overproduction strains before and at different time points up to 2 h after exposure of the cells to 50 mM acetic acid (pH 4.5). Our data shows similar *HAA1* transcript levels in the Haa1 wild-type and mutant strains, while an about 1.5-fold increase was shown in the Haa1 overproduction strain (Fig. 5). No detectable fluctuation in *HAA1* transcript levels was found in any of the Haa1 mutant strains as compared to the Haa1 wild-type strain during a period of up to 2 h after exposure of the cells to acetic acid.

In order to investigate whether the expression of the mutant *HAA1* alleles as well as the overexpression of *HAA1* result in an increased transcription of acetic acid-responsive genes of the Haa1-regulon, the transcript levels from four of these genes (*TPO2*, *TPO3*, *YRO2*, and *YGP1*) [7, 19] were determined during the first 2 h following sudden exposure to acetic acid (Fig. 5). *FPS1* was used as a negative control in the experiment, given that the gene is a determinant of *S. cerevisiae* susceptibility to acetic acid [38] and that its expression has been shown to be independent of Haa1 [15]. The latter is supported by our data, which shows that *FPS1* transcript levels are maintained constant during acetic acid exposure and no significant differences are detected among the strains (Fig. 5). With regard to the genes *TPO2*, *TPO3*, *YRO2*, and *YGP1*, transcript levels were clearly increased in strain CEN.PK113-7D Haa1^OE^, correlating with the increased *HAA1* mRNA abundance in this strain; however, a similar increase was also observed in strains CEN.PK113-7D Haa1^S135F_Truncated^ and Haa1^S135F^ (Fig. 5). It is also noteworthy that no detectable strain-specific differences in the low basal transcription level of the respective target genes before acetic acid addition were found, and that no significant transcriptional activation of the four selected Haa1-target genes was registered in the *haa1* deletion mutant (Fig. 5). Remarkably, the level of transcriptional activation from all selected Haa1-target genes by Haa1 wild-type and mutant proteins correlates with the level of acetic acid tolerance exhibited by the corresponding strains (Fig. 5).

## Discussion

Previous studies have shown that the acetic acid tolerance of *S. cerevisiae* strains S288c and Ethanol Red can be improved by exchanging the native *HAA1* promoter for the strong and constitutive *TDH3* promoter [15, 16, 39]. This overexpression of *HAA1* was shown to result in an increased transcript level of Haa1-target genes *TPO2, TPO3, YRO2* and *YGP1* even in the absence of acetic acid [15], implying that the higher Haa1 protein level resulted in a constitutive activation of the Haa1-regulon. It is assumed that the latter better arms the cells once they are exposed to the acid. In the current study, overexpression of *HAA1* was achieved by introducing an additional copy of the *HAA1* coding sequence under the control of the endogenous *HAA1* promoter and terminator. This engineered strain showed an about 1.5-fold increase in *HAA1* transcript level in unstressed cells, which is in clear contrast to the Haa1 overproduction strain constructed by Inaba et al. [16] that showed a sevenfold increase. The strain constructed in the current study did also not show an increase in the expression level of tested Haa1-target genes in the absence of acetic acid which is in contrast to what has been observed in the study of Tanaka et al. [15]. Although it remains to be clarified whether an even higher expression of *HAA1* in the CEN.PK background could further increase its acetic acid tolerance, we assume that a higher expression rather represents a burden to the cells. In fact, the studies of Tanaka et al. [15] (2.5-fold increase) and Inaba et al. [16] (sevenfold increase) recognized a reduction in growth under non-stress conditions, which would be a disadvantage for industrial applications and suggests a serious limitation of cell endurance against acetic acid. Notably, the Haa1 overproduction strain constructed in the current study did not show any growth deficit in the absence of acetic acid (data not shown). It was also interesting to see that the Haa1 overproduction strain constructed in the current study showed a higher fraction of cells resuming proliferation in the presence of acetic acid as compared to the wild-type strain. To the best of our knowledge, this has been the first time that a positive impact of an engineered Haa1 variant or of an increased cellular Haa1 abundance on this parameter has been measured. In order to understand this phenomenon at the molecular level, respective studies at the single-cell level are envisaged.

The isolation of mutant *HAA1* alleles that significantly improve acetic acid tolerance demonstrates the success of engineering a regulon-specific transcription factor to improve a complex phenotype that is controlled by this regulon. In fact, the strains CEN.PK113-7D Haa1^S135F^ and CEN.PK113-7D Haa1^S135F_Truncated^ showed an acetic acid tolerance that was similar or even slightly higher than the strain CEN.PK113-7D Haa1^OE^. Interestingly, in contrast to the Haa1 overproduction strain, the Haa1 mutant strains showed an improved acetic acid tolerance that was not concomitant with an increased *HAA1* transcript level. Hence, the question arises why the different genetic modifications and underlying molecular mechanisms observed in the Haa1 mutant and overproduction strains can result in the same tolerance phenotype. In principle it is possible that a higher stability of the mutant Haa1 proteins, in particular of the truncated variant, may underlie their increased activity. We tried to test this hypothesis by quantitative immunodetection of Haa1 proteins using the constructed Haa1-GFP fusion proteins and antibodies against GFP (data not shown). However, the naturally low cellular levels of the transcription factor did not allow an unambiguous quantification of the different protein variants.

In addition to an altered protein stability, it is also conceivable that the mutant Haa1 proteins may exhibit different structures that improve their ability to recruit the transcription machinery to the target genes of the Haa1-regulon. According to the PhosphoGRID database, the critical point mutation identified in our study (S135F) substitutes a serine residue located within a putative kinase target site [40]. It is likely that this mutation influences the phosphorylation level and consequently biological activity of the Haa1 protein, considering that the position of this residue is relatively close to the DNA-binding domain.

It is also noteworthy that the loss of one-third of the entire Haa1 sequence (the last 212 C-terminal residues) leads to a small, although significant increase of the protein biological activity. Remarkably, a recent study identified acetic-acid tolerant mutant *HAA1* alleles that exhibit multiple mutations in the transactivation domain, mainly in the end of the C-terminal region (mutant 2: F440Y, P518S, I591V, H605Y, S622F, S639F, S673L; and mutant 40: D508Y, N510K, A527V, N554Stop) [41], while another study identified the single mutation S506N as contributing to the difference in acetic acid tolerance between a strain with unusually high tolerance and an industrial reference strain [42]. With the exception of the F440Y mutation, all identified mutations are located in the sequence that was lost in the Haa1^Truncated^ protein identified in the current study. Although little is known about the structure of the Haa1 transactivation domain, our results suggest that the C-terminal region of the Haa1 sequence may harbor domains involved in the negative regulation of the protein’s activity. Nevertheless, full understanding of the mechanisms underlying the different biological activity of the mutant *HAA1* alleles identified will require additional work, including work on the structural characterization of the protein-DNA complexes.

## Conclusion

In the current study, mutant *HAA1* alleles have been identified that improve the acetic acid tolerance of the strain CEN.PK113-7D, representing a prototrophic version of the industrially-relevant *S.* *cerevisiae* CEN.PK family. Initial investigations with regard to the biochemical mechanisms underlying the improved phenotype suggests that the alleles encode proteins that are more active compared to the wild-type protein. The mutant *HAA1* alleles could be used for the construction of robust strains for biotechnological processes using lignocellulosic hydrolysates as feedstock, particularly since the reverse engineered strains did not show any impairment with regard to growth under non-stress conditions. The alleles may also inspire further studies to better understand the underlying molecular basis for the increased activity of the respective proteins in inducing the Haa1-regulon.
